# Wavelength-dependent reactivity of germanium-based photoinitiators

**DOI:** 10.1007/s00706-025-03379-5

**Published:** 2025-09-17

**Authors:** Max Schmallegger, Dmytro Neshchadin, Hilde Freißmuth, Thomas Lainer, Mathias Wiech, Georg Gescheidt, Michael Haas

**Affiliations:** 1 Institute of Physical and Theoretical Chemistry, Graz University of Technology, Graz, Austria; 2 Institute of Inorganic Chemistry, Graz University of Technology, Graz, Austria

**Keywords:** Photoinitiators, Photochemistry, Polymerization, Acylgermanes

## Abstract

This study investigates the wavelength-dependent reactivity of the three germanium-based type I photoinitiators bis(4-methoxybenzoyl)diethylgermane (Ivocerin®), tris(2,4,6-trimethylbenzoyl)ethylgermane, and tetrakis(2-methylbenzoyl)germane. By systematically varying LED wavelengths from 365 to 495 nm, we quantify quantum efficiencies/absorbance and correlate them with the photobleaching behavior and penetration depths. The results highlight the crucial relationship between the emission profile of the light source and the efficiency of the photoinitiators. These insights are particularly relevant for optimizing light-driven processes in applications such as biomedical printing, adhesives, and spatially controlled polymerization.

**Graphical Abstract:**

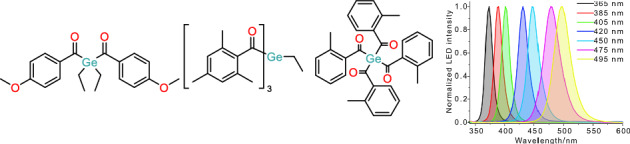

**Supplementary Information:**

The online version contains supplementary material available at 10.1007/s00706-025-03379-5.

## Introduction

Photopolymerization has become a cornerstone technology across diverse fields, including coatings, electronics, and additive manufacturing [1–3]. Central to this process are photoinitiators – molecules that absorb light and generate reactive species initiating polymerization. Among them, type I photoinitiators (shown for an acylgermane in Scheme 1) undergo photo-induced unimolecular *a-*cleavage in the triplet state (via an initial singlet state and intersystem crossing) providing radicals (Ac• and Ge•), which add to double bonds [4].

**Figure:**
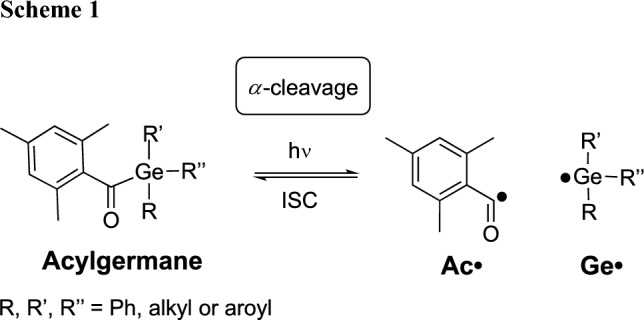

This intrinsic efficiency, particularly under mild conditions, has made type I systems highly attractive for high-resolution and rapid-curing applications. A major advantage of this approach is the reduced environmental impact due to the absence of volatile organic compounds (VOCs), along with rapid curing at ambient temperatures and low energy requirements [5].

Historically, most type I systems were designed to absorb in the ultraviolet (UV) range, where photon energies are sufficient to promote electronic excitation and bond cleavage [6]. However, the use of UV light presents significant limitations, including shallow curing depths due to strong absorption and scattering, and the potential for DNA damage and cytotoxicity in biological applications [7].

Consequently, recent advances in type I photoinitiators have focused on addressing these longstanding limitations, such as toxicity, poor solubility in aqueous media, and narrow absorption windows. Moreover, driven by the expanding demands of biocompatible materials and digital light processing, there has been a notable shift toward initiators that are compatible with readily available LED light sources.

Innovations in molecular design, such as the incorporation of heavy atoms, and extended π-conjugation, have yielded initiators with enhanced photoreactivity and visible-light absorption. In this context, tailored synthesis of acylphosphine oxides [8–11], acylgermanes [12–18], and other metalloid-containing compounds has led to new classes of photoinitiators with tunable photophysical and redox properties [19–21]. These developments have expanded the applicability of type I systems beyond traditional UV-based curing.

Since many of these tailored photoinitiators have broad absorption bands, their reactivity is not uniform across all wavelengths; instead, it depends heavily on their specific absorption characteristics and the energy of the incident light [6, 7].

**Fig. 1:**
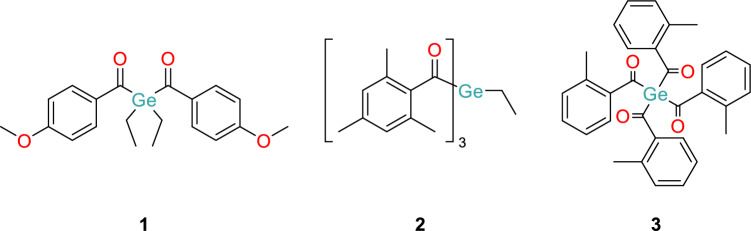
Investigated acylgermanes

Given our recent efforts in the synthesis and photochemical characterization of acylgermanes, we set out to investigate the wavelength-dependent reactivity of three acylgermanes, bis(4-methoxybenzoyl)diethylgermane (**1**, Ivocerin®), tris(2,4,6-trimethylbenzoyl)methylgermane (**2**), and tetrakis(2-methylbenzoyl)germane (**3**) (Fig. 1).

These compounds were chosen based on their distinct absorption profiles and relevance to visible-light applications in technology and biomedical applications. Upon exposure to varying wavelengths, each photoinitiator displayed markedly different reactivity, underscoring the importance of spectral compatibility between the initiator and the Light source. By selecting photoinitiators with reactivity tailored to particular wavelengths, researchers and engineers can improve the efficiency, depth control, and precision of Light-induced reactions. This wavelength-dependent behavior is also vital for minimizing undesired side reactions, reducing energy consumption, and enabling the use of specific light sources such as LEDs or lasers. As such, it represents a key factor in the rational design and performance optimization of modern light-based technologies, particularly those requiring spatial and temporal control, such as biomedical 3D printing, microstructuring, and photopatterning.

## Results and discussion

**Fig. 2:**
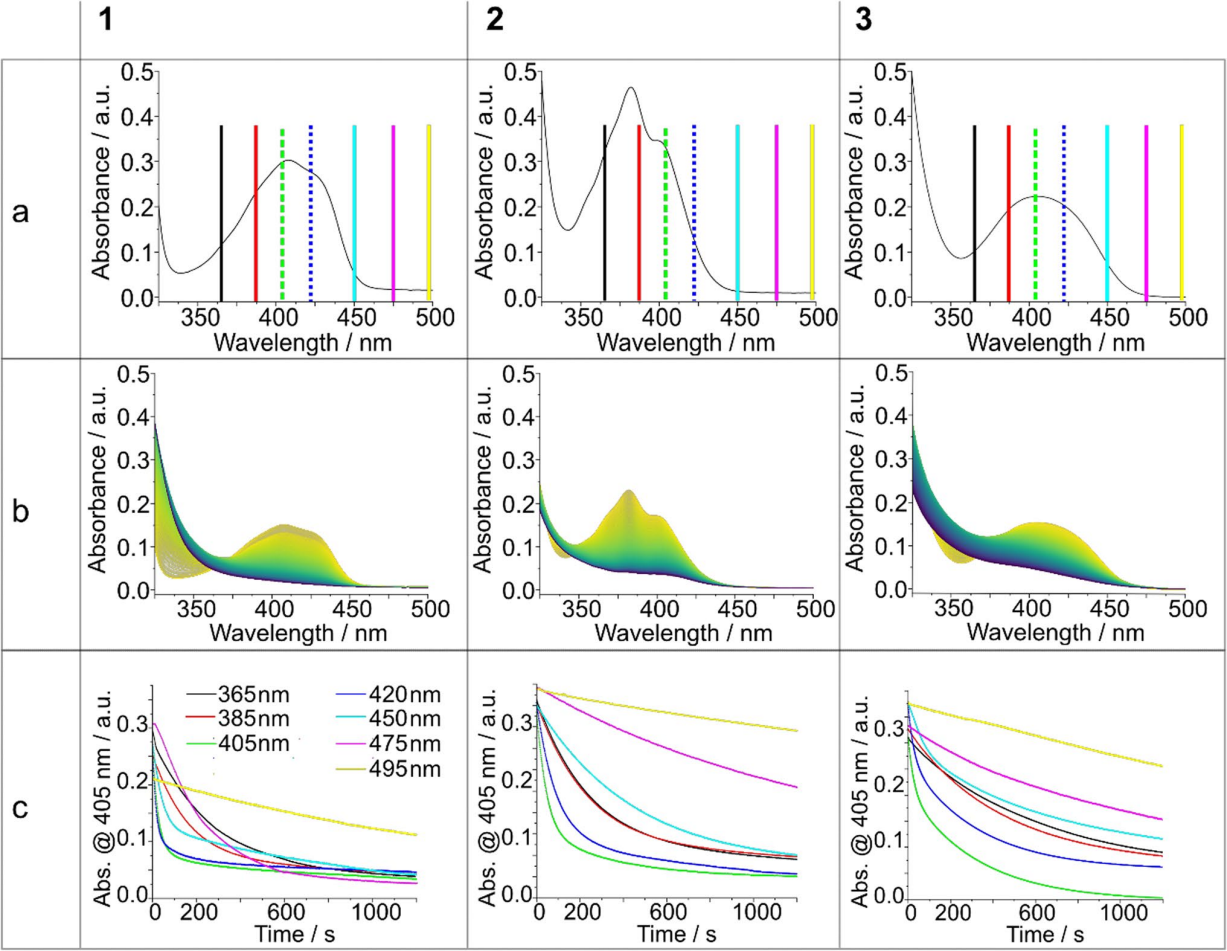
**a** UV–Vis spectra of **1–3**, the vertical lines indicate the irradiation wavelengths; **b** Corresponding photobleaching at 365 nm irradiation (remaining spectra, see Supporting Information); **c** Time-dependent decay curves at all seven wavelengths, see color codes in Fig. 2a

**Fig. 3:**
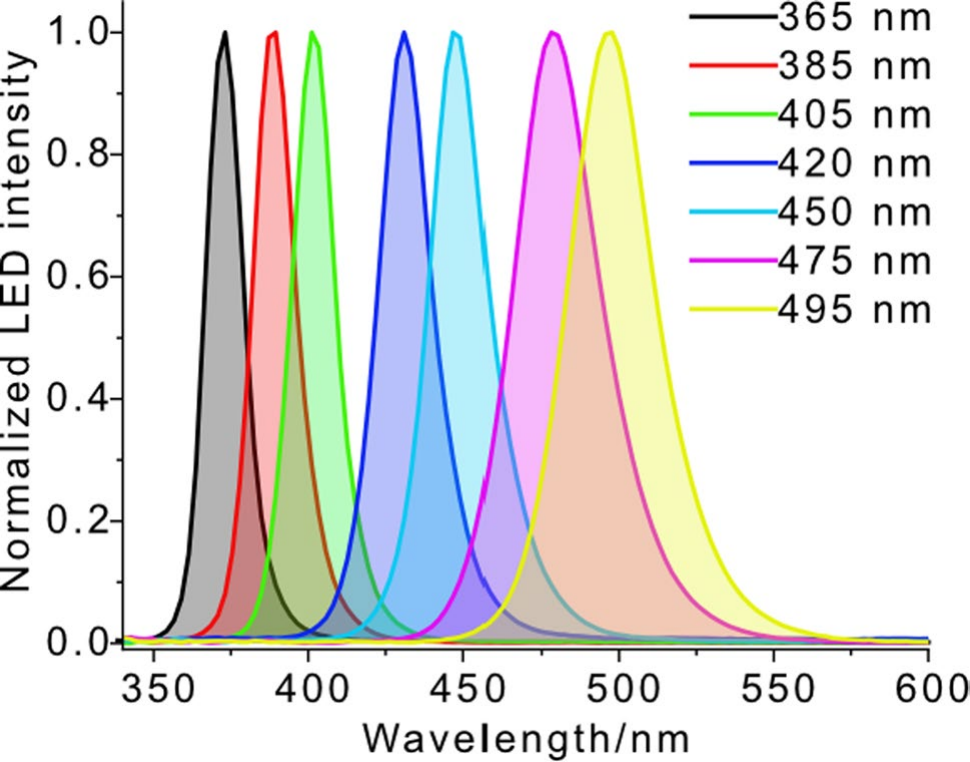
Light profiles of LEDs (peaks at 365, 385, 405, 420, 450, 475, and 495 nm) used in this investigation

In their UV–Vis spectra, acylgermanes **1**, **2**, and **3** reveal absorption bands below approximately 360 and between 370 and 480 nm (Fig. 2a). The latter, weaker broad bands are based on *n*-π* transitions. Excitation of these latter bands leads to a weakening of the Ge–C(O) bond and, finally, *a-*cleavage (Scheme 1). Accordingly, we have photolyzed acylgermanes **1, 2,** and **3** using LEDs in the range covering these bands emitting at 365, 385, 405, 420, 450, 475, and 495 nm (see Fig. 3). We employed both low- and high-power LEDs to ensure the broad applicability of our results. In parallel, we recorded time-resolved UV–Vis spectra (Fig. 2c).

In the *n*-π* range, **1** reveals a slightly structured band with relative maxima at 410 and 430 nm, **2** shows two maxima at 385 and 405 nm, and **3** shows one at 410 nm. The absorption spectra of **1, 2,** and **3** fade out at 460, 450, and 480 nm, respectively (Fig. 2a). When considering the wavelength-dependent photobleaching (Fig. 2b), the emission spectra of the applied LED light sources must be considered. The corresponding emission profiles are presented in Fig. 3.

When evaluating wavelength-dependent efficiencies, it is important to recognize that, although the emission wavelength of an LED is nominally well-defined, the emitted radiation is not strictly monochromatic. Depending on the peak (or dominant) wavelength, the spectral bandwidth of LEDs can be substantial—up to approximately 100 nm for a 495 nm LED, and around 50 nm for a 365 nm LED (measured at the base of the emission profile). As a result, irradiation with a 495 nm LED can also excite absorption bands around 450 nm, albeit with reduced intensity (Fig. 3).

**Fig. 4:**
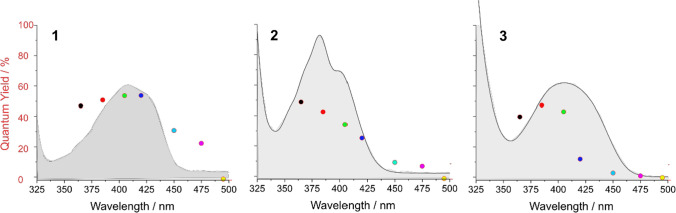
Wavelength-dependent quantum efficiency of **1, 2,** and **3**. The corresponding UV–Vis spectra are presented as the grey shape

Bearing this in mind, we can now consider the wavelength-dependent quantum efficiency for the *α*-cleavage in molecules **1–3** shown in Fig. 4.

The colored dots in Fig. 4 represent the quantum efficiency obtained upon the use of the LEDs with peaks at 365, 385, 405, 420, 450, 475, and 495 nm. Connected with the emission line widths presented in Fig. 3, the quantum efficiencies generally relate to the absorbance at which the LED emits. However, it must be borne in mind that the spectral width of the emission lines is rather broad, which explains why photo-activity is observed even when the applied LED has a nominal wavelength in a range at which the substrate does not absorb (up to 50 nm away from its absorption).

**Fig. 5:**
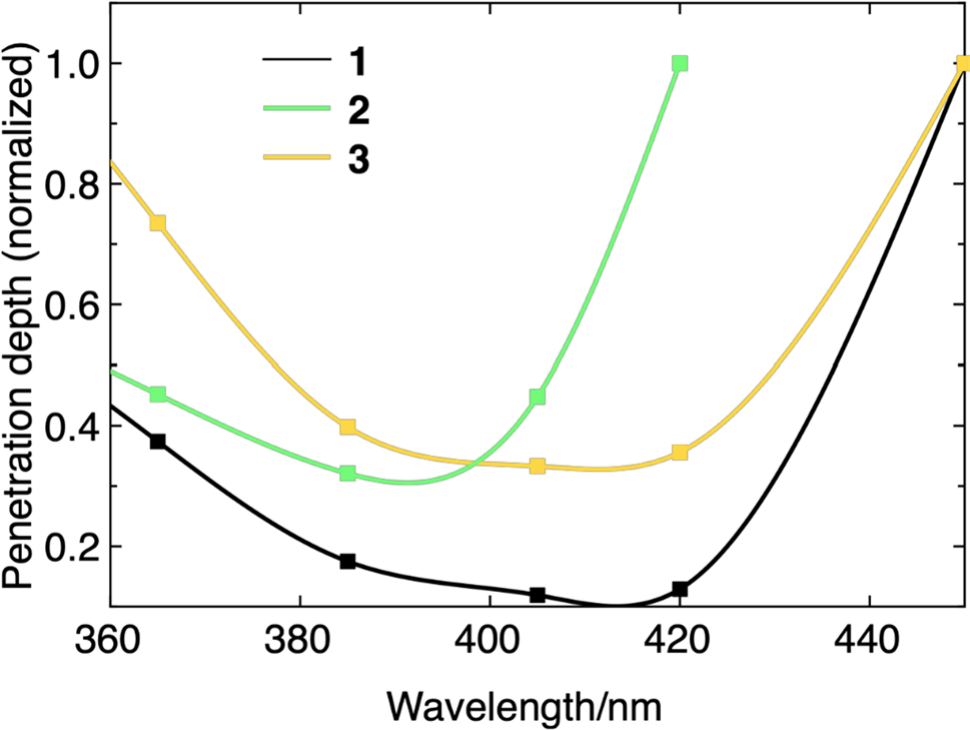
Relative wavelength-dependent penetration depths (normalized, with a remaining intensity of 37%) for **1, 2,** and **3**

The amount of absorbance and the quantum efficiency, however, are not per se limiting factors for using a wavelength that only matches a region of low absorbance. Figure 5 represents the calculated relative penetration depths for **1, 2,** and **3**. For this representation, we have chosen to use percentages of penetrations instead of absolute values (which depend on concentrations). This provides a more general perception of the interplay of absorbance, quantum efficiency, and penetration depth. On one hand, lower absorption produces fewer initiating radicals (see Scheme 1, at a lower rate) for the polymerization, but it allows to cure thicker layers.

### Structure—property discussion

To fully understand the wavelength-dependent reactivity observed for the three acylgermane photoinitiators, it is essential to consider their distinct structural features and how these influence photophysical properties. Ivocerin® (**1**), with two *para*-methoxybenzoyl and two ethyl substituents at germanium, benefits from strong electron-donating methoxy groups that extend conjugation and red-shift absorption into the visible range. This results in relatively broad absorption centered around 410 and 430 nm and a tail extending to approximately 460 nm, enabling effective activation under blue LEDs. Its strong absorption, however, comes at the cost of Limited penetration depth, which restricts its use to thin-layer applications but favors high-resolution surface curing, which is valuable in 3D printing or coating technologies.

In contrast, tris(2,4,6-trimethylbenzoyl)ethylgermane (**2**) carries three mesitoyl groups, whose *ortho*-methyl substituents induce substantial steric hindrance. This perturbs the conjugation between the carbonyl group and the aromatic ring, resulting in a hypsochromic shift compared to compound 1. Accordingly, its absorption peaks are observed at shorter wavelengths (385 and 405 nm), with a rapid drop-off beyond 450 nm. Despite this blue shift, compound 2 exhibits relatively high quantum efficiencies at shorter wavelengths, particularly under UV to violet excitation. Thus, it is well-suited for fast radical generation under high-energy irradiation, though its limited penetration depth makes it less ideal for thick-layer curing.

Tetrakis(2-methylbenzoyl)germane (**3**) presents a different case: it contains four *ortho*-methylbenzoyl groups that, while sterically bulky, lack the extended conjugation or strong resonance-donating effects of the para-methoxy substituents in 1. Nevertheless, the presence of four chromophores produces a cumulative effect, broadening the absorption and allowing activation at longer wavelengths. With an absorption profile that extends past 480 nm and measurable quantum efficiency even at 475 and 495 nm, compound **3** offers the broadest operational window among the three initiators. Although it generates radicals with lower efficiency, its weak absorbance in the blue-green range allows for significantly greater penetration depth, making it particularly attractive for curing thick layers or for use with low-energy visible-light sources, which is a feature especially important for biocompatible and spatially controlled applications.

## Conclusion

Our investigation demonstrates the importance of considering the wavelength of the chosen light source in relation to the absorption spectrum, absorbance, and quantum efficiency of the photoinitiator. Even a photoinitiator with low quantum efficiency can effectively initiate photopolymerization in thick layers, provided that a sufficiently high-intensity LED is used. This compensates for the limited efficiency and still enables effective curing. Evaluating the resulting polymer properties, such as mechanical strength, when using LEDs of different wavelengths represents a logical and valuable direction for further study. Overall, the different photoinitiators illustrate how subtle structural changes dramatically influence absorption characteristics, quantum yields, and practical curing performance.

## Experimental

Ivocerin (**1**) was obtained from Ivoclar Vivadent. Tris (2,4,6-trimethylbenzoyl)methylgermane (**2**) and tetrakis(2-methylbenzoyl)germane (**3**) were synthesized as described previously [22, 23]. Acetonitrile (Riedel-de Haën) was obtained at the highest purity available and employed as received.

### Steady-state photolysis

UV–Vis spectra were recorded on a UV–Vis spectrometer equipped with optical fibres and a 1024-pixel diode-array detector (J&M Analytik AG, Essingen, Germany). Standard fluorescence quartz cuvettes were used for all measurements. The concentration of **1, 2,** and **3** in acetonitrile was around 0.3 mM for all measurements.

Sample illumination was performed perpendicular to the spectrometer beam during the time-resolved measurements. The sample was stirred during measurement to provide a homogeneous solution.

The LED output powers at 365, 385, 405, 420, 450, 475, and 495 nm were 3, 3.5, 20, 25, 25, 40, and 70 mW, respectively. The optical output power of the LEDs was measured using a calibrated spectrophotometer (GL Spectis 1.0, GL Optic Lichtmesstechnik GmbH, Weilheim, Germany) equipped with an integrating sphere. The LEDs were placed in front of the sphere aperture (diameter = 9 mm), resembling the geometry of the spectroscopic setup. LEDs were purchased from Roithner LaserTechnik GmbH (Vienna, Austria).

Quantum yields were determined according to the procedure described in the literature [24].

## Supplementary Information

Below is the link to the electronic supplementary material.

Supplementary file1 (DOCX 4510 KB)

## Data Availability

All data are included in the manuscript. There is no need for a data statement.
