# Supplementary material for: Wavelength-dependent reactivity of germanium-based photoinitiators
Source: Monatsh Chem. 2025 Sep 17;157(3):327–32. doi: 10.1007/s00706-025-03379-5 (PMC12946342; doi:10.1007/s00706-025-03379-5)
Supplement: Supplementary file 1 — Supplementary file1 (DOCX 4510 KB) [file 706_2025_3379_MOESM1_ESM.docx]

SUPPORTING INFORMATION

Wavelength-Dependent Reactivity of Germanium-Based Photoinitiators

**Max Schmallegger^1^ ● Dmytro Neshchadin^1^ ● Hilde Freißmuth^1^ ● Thomas Lainer^2^ ● Mathias Wiech^1^ ● Georg Gescheidt^1^● Michael Haas^2^**

1. Time-resolved Bleaching spectra providing the data for Fig.2 for **1**, **2**, and **3**................. 2-4

2. Calculation of penetration depth.................................................................................... 5


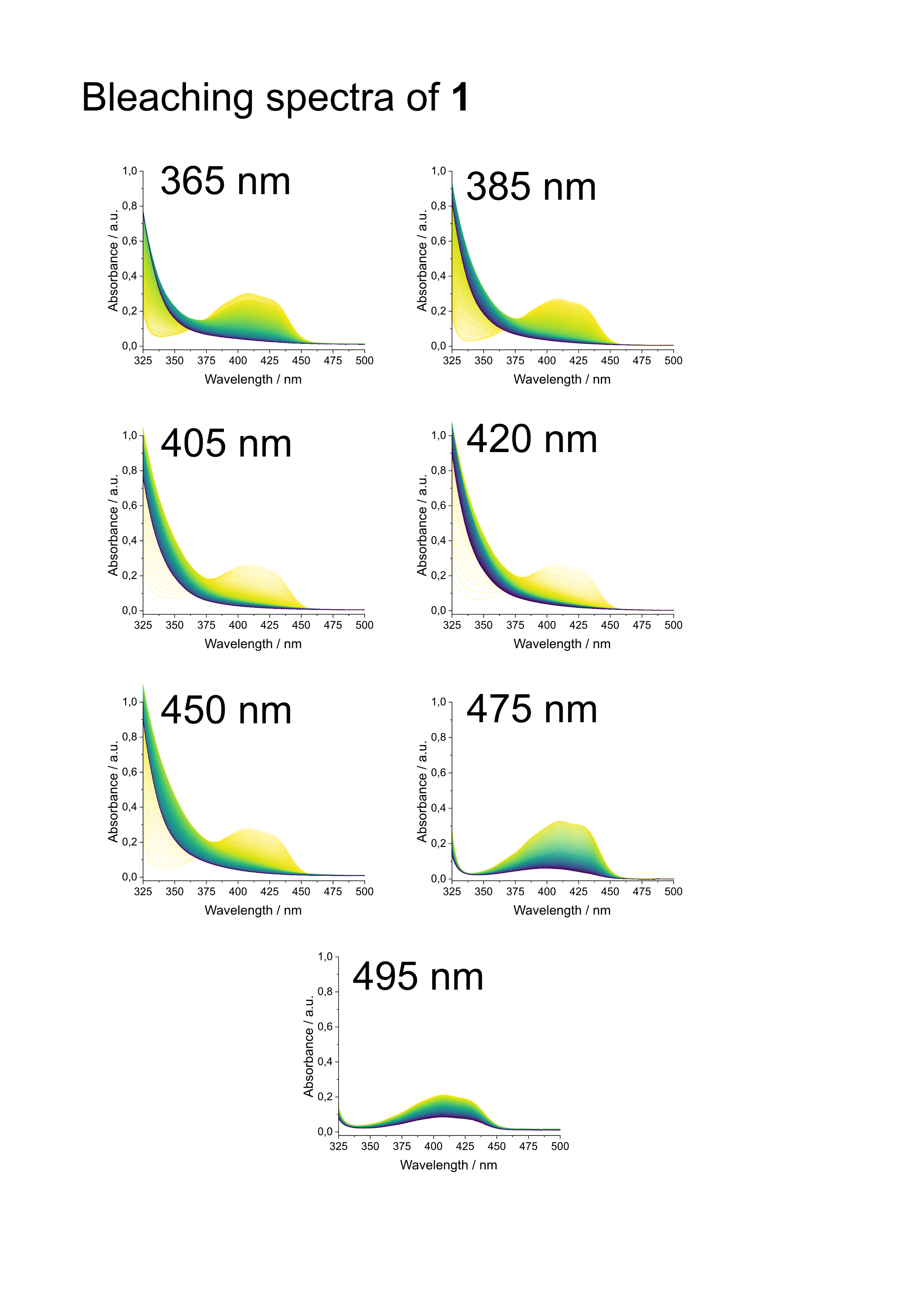


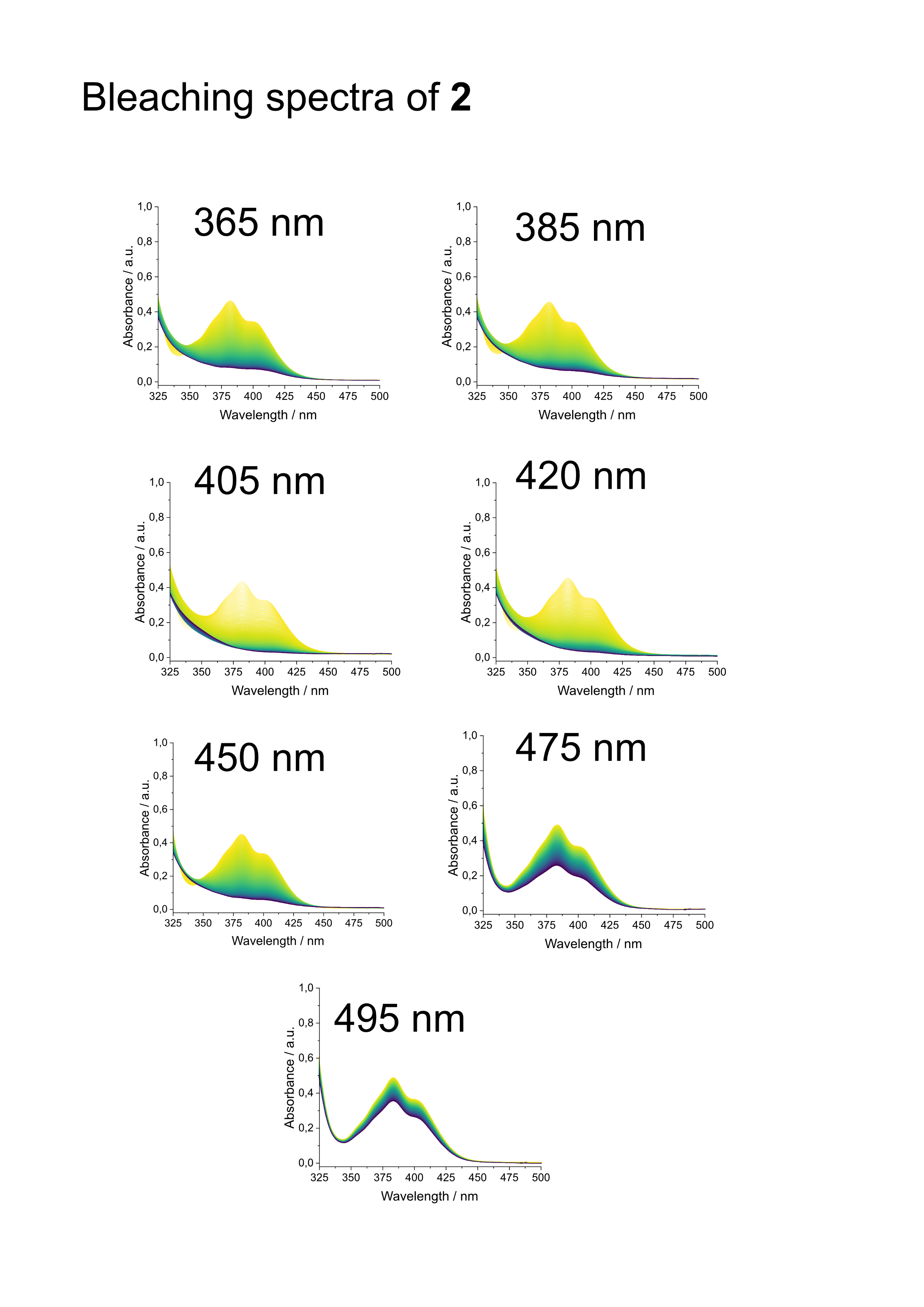


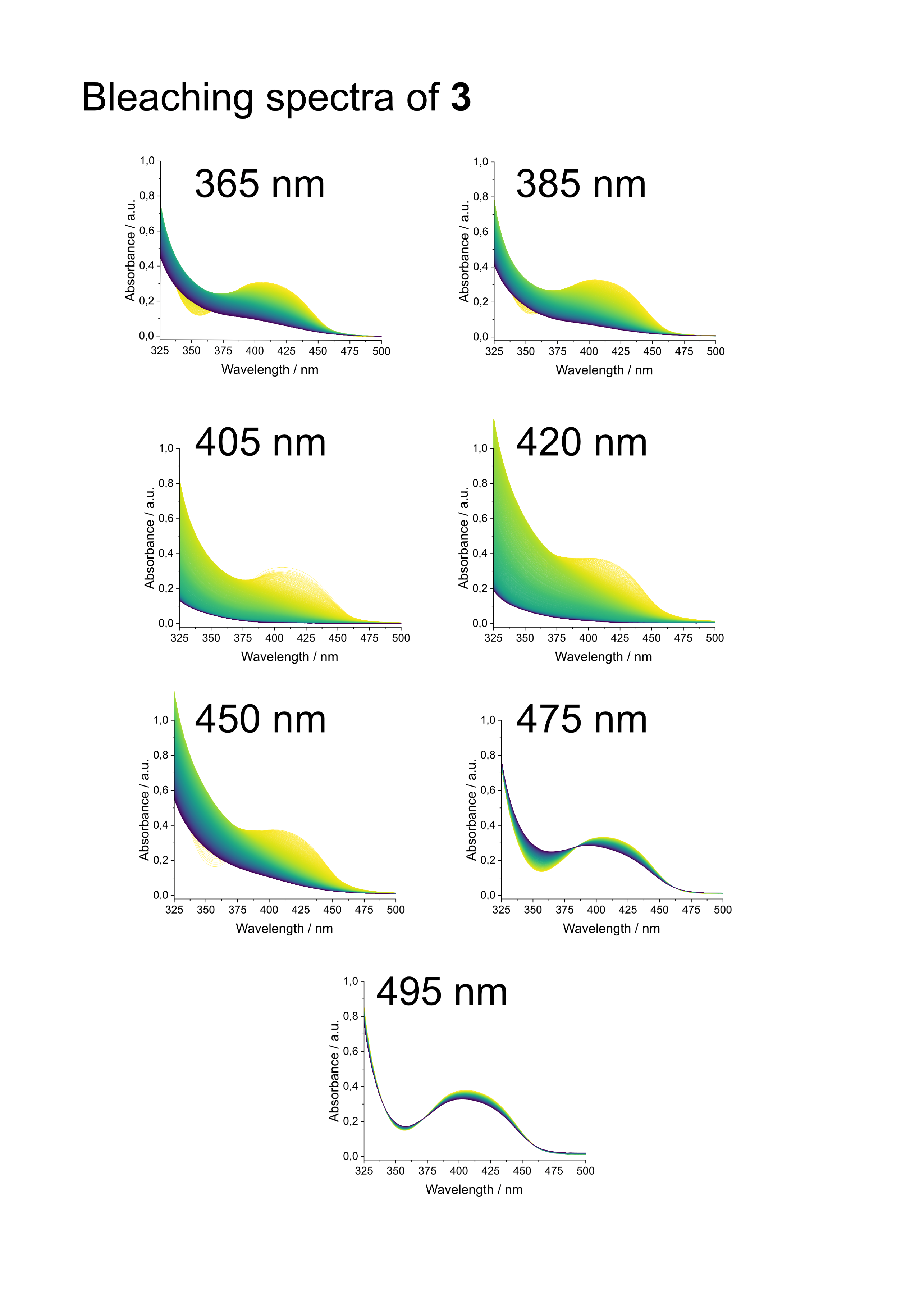


*For the calculation of the penetration depth* d, we use the Beer-Lambert law:

*I* = *I*_0_ · 10*^ε c d^*

For a remaining intensity of 37% (which corresponds to 1/*e*) :

*I* = 0.37 · *I*_0_

accordingly:

*d* = log(0.37)/*e* (with *c* = 1)
